# Binding Cooperativity Matters: A GM_1_-Like Ganglioside-Cholera Toxin B Subunit Binding Study Using a Nanocube-Based Lipid Bilayer Array

**DOI:** 10.1371/journal.pone.0153265

**Published:** 2016-04-12

**Authors:** Nolan C. Worstell, Pratik Krishnan, Joshua D. Weatherston, Hung-Jen Wu

**Affiliations:** Department of Chemical Engineering, Texas A&M University, College Station, Texas, United States of America; University of British Columbia, CANADA

## Abstract

Protein-glycan recognition is often mediated by multivalent binding. These multivalent bindings can be further complicated by cooperative interactions between glycans and individual glycan binding subunits. Here we have demonstrated a nanocube-based lipid bilayer array capable of quantitatively elucidating binding dissociation constants, maximum binding capacity, and binding cooperativity in a high-throughput format. Taking cholera toxin B subunit (CTB) as a model cooperativity system, we studied both GM_1_ and GM_1_-like gangliosides binding to CTB. We confirmed the previously observed CTB-GM_1_ positive cooperativity. Surprisingly, we demonstrated fucosyl-GM_1_ has approximately 7 times higher CTB binding capacity than GM_1_. In order to explain this phenomenon, we hypothesized that the reduced binding cooperativity of fucosyl-GM_1_ caused the increased binding capacity. This was unintuitive, as GM_1_ exhibited higher binding avidity (16 times lower dissociation constant). We confirmed the hypothesis using a theoretical stepwise binding model of CTB. Moreover, by taking a mixture of fucosyl-GM_1_ and GM_2_, we observed the mild binding avidity fucosyl-GM_1_ activated GM_2_ receptors enhancing the binding capacity of the lipid bilayer surface. This was unexpected as GM_2_ receptors have negligible binding avidity in pure GM_2_ bilayers. These unexpected discoveries demonstrate the importance of binding cooperativity in multivalent binding mechanisms. Thus, quantitative analysis of multivalent protein-glycan interactions in heterogeneous glycan systems is of critical importance. Our user-friendly, robust, and high-throughput nanocube-based lipid bilayer array offers an attractive method for dissecting these complex mechanisms.

## Introduction

Glycan binding proteins (GBPs) often recognize glycans present on cell surfaces via multivalent binding mechanisms. Many GBPs contain multiple glycan binding subunits that bind to multiple glycans attached to lipids or membrane proteins on cell surfaces. These glycans can freely diffuse and rotate on a 2D fluidic cell membrane, enabling self-organization for multivalent interactions with GBPs. Such multivalent interactions are mediated by cooperative effort between glycan-bound subunits that influences binding avidity and/or specificity[1]. A good example of this cooperative binding is the interaction of cholera toxin B subunit (CTB) with gangliosides. CTB is a homopentamer that strongly associates with GM_1_ gangliosides. Positive cooperativity between bound GM_1_ molecules can raise CTB-GM_1_ binding avidity by several orders of magnitude [2, 3]. The CTB-GM_1_ stepwise binding mechanism has been studied by isothermal titration calorimetry (ITC) and mass spectrometry (MS) [4, 5]. In one study, Klassen and coworkers observed that the binding affinity (association constant) of the unbound subunit doubles in value when a bound GM_1_ is adjacent to the unbound pocket, demonstrating the positive cooperativity of GM_1_-CTB binding [5]. Furthermore, this concept of binding cooperativity has been widely utilized to design high affinity inhibitors for various multivalent GBPs, including biotoxins and lectins [6].

Due to its high GM_1_ binding avidity, CTB has been widely used to monitor the quantity and localization of GM_1_ in cell staining [7, 8]. However, Yanagisawa et al. observed CTB could bind to cell surfaces in the absence of GM_1_ gangliosides [9]. They hypothesized that CTB binding to mouse embryonic neuroepithelial cells could be caused by the other GM_1_-like gangliosides, including fucosyl-GM_1_. However, the mechanism is still not clear and requires a vast quantity of cross-reactivity data to elucidate. In order to quantify the cross-reactivity between CTB and the mixed gangliosides, a high-throughput, easy-to-use, and robust analytical tool is of critical importance.

The typical tool for glycan recognition is the glycan microarray where various synthetic or natural glycans are immobilized on a solid surface [10, 11]. In this technique, bound analytes are detected by labeling techniques, such as fluorescent and immunostaining assays, or by label-free detection technologies that require special instrumentation [10]. A limitation of current glycan microarray technologies is the required immobilization of glycan receptors onto the substrate. This creates a problem because immobilized glycans cannot completely achieve multivalent binding. It is impossible to control the spacing and orientation of glycans to match precisely the configuration of binding pockets in the target GBPs. Hence, the presentation of glycans on microarray surfaces, including linker effects and glycan density, influences GBP binding[11]. This intrinsic drawback limits the ability of glycan microarrays to quantify the complex multivalent interactions. To overcome this drawback, an alternative approach is to insert glycans (e.g. glycolipids or neoglycolipids) into fluidic bilayers instead of immobilizing them onto a substrate [4, 12–15]. In a fluidic bilayer system, glycans can freely move and encounter target GBPs to enable multivalent interactions. Although the fluidic bilayer array format has been demonstrated by different research groups [13, 14, 16–18], none of these techniques have become widely spread throughout the biological sciences. This is probably due to a lack of accessibility and flexibility.

**Fig 1:**
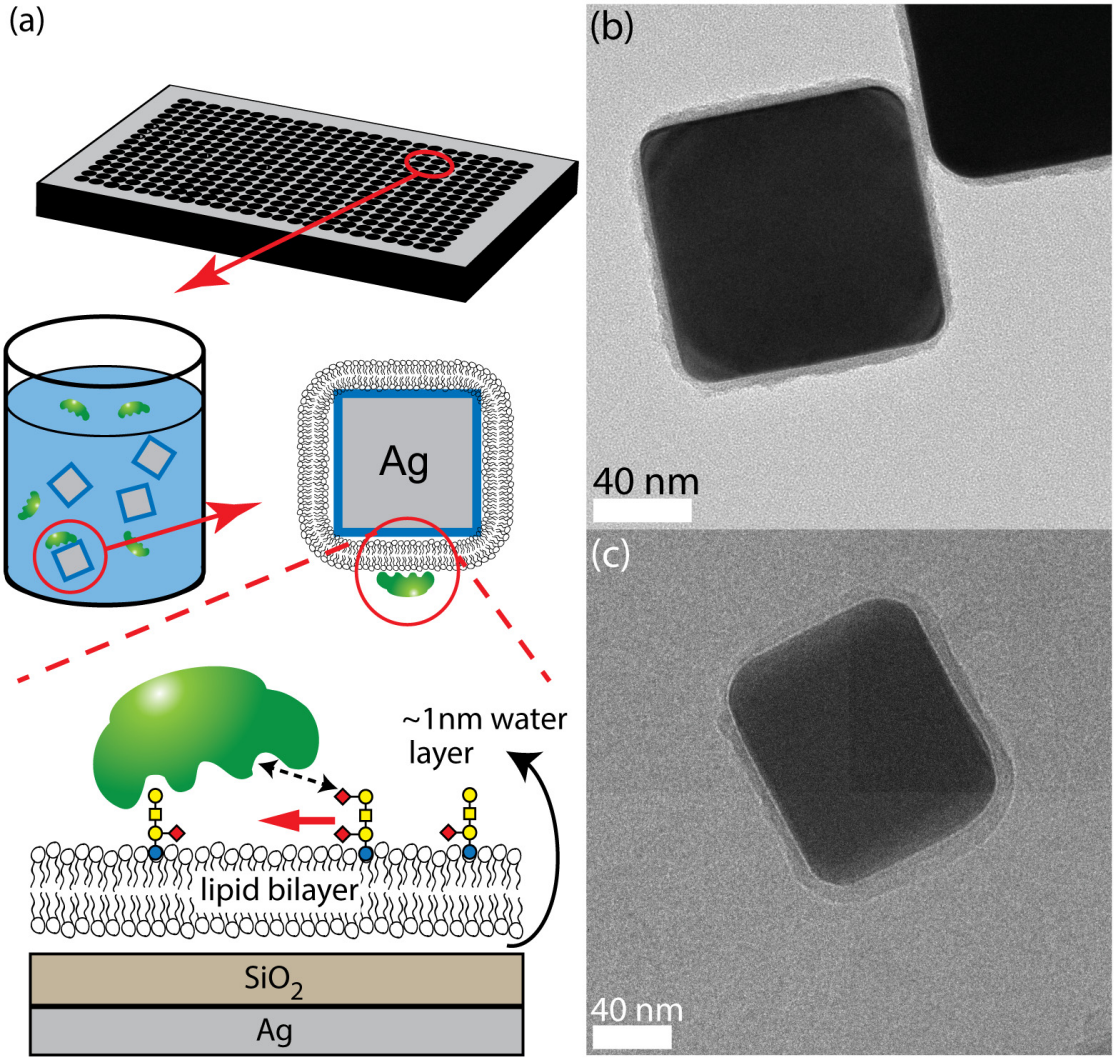
Overview schematic of nanocube-based sensor with confirmation by TEM. (a) A schematic of the nanocube-based lipid bilayer array. Silica coated silver nanocubes (Ag@SiO_2_ nanocubes) are covered by a supported fluidic lipid bilayer that incorporates gangliosides. Equilibrium binding was detected in a 384 well plate by monitoring the extinction spectra in a microplate spectrophotometer. (b) A TEM image of the silica shell coated onto the Ag nanocubes. (c) A cryo-TEM image of the supported lipid bilayer coated onto the Ag@SiO_2_ nanocubes.

To address prior systems’ lack of accessibility and flexibility, we introduced a unique nanocube sensor for direct measurements of CTB binding onto lipid bilayer surfaces [19] (Fig 1a). This Ag@SiO_2_ core-shell nanocube sensor enables label-free detection of protein binding to lipid bilayer surfaces by taking advantage of the fluidic bilayer system. To create the fluidic bilayer, a thin water layer mediates the supported lipid bilayer’s interaction with the sensor’s silica surface. This thin layer provides a flexible buffer that enables the bilayers to mimic an idealized cell membrane and possess similar two-dimensional fluidity. Protein binding to lipid bilayers is monitored by observing the extinction spectra shift of the localized surface plasmon resonance (LSPR) using a standard UV-Vis spectrophotometer. Our previous study has demonstrated the application of this nanocube sensor [19]. The advantages of this platform are (1) high accessibility (only requiring standard spectrophotometer), (2) ease of use (simple “mix-and-then-detect” protocol), (3) high flexibility (allowing end users to build their own assays in-house without special equipment), and (4) label-free detection. In contrast to the conventional labeling techniques, this label-free sensor can directly quantify absolute surface densities of bound proteins without calibration prior to binding measurements. These outstanding features enable the quantitative analysis of GBP binding mechanisms.

In this study, we improved the prior nanocube-based sensing platform to enable high-throughput detection. We successfully achieved large-scaled synthesis of high quality nanocube sensors (one synthesis batch allowing up to 20,000 measurements), and adapted the sensors to a high-throughput microplate reader (384 well plate). This novel nanocube-based lipid bilayer array allowed us to simultaneously perform many CTB binding measurements at different experimental conditions, facilitating the dissection of complex binding mechanisms. In this article, we measured the cross-reactivity of CTB with various gangliosides, including GM_1_, GM_2_, and fucosyl-GM_1_. We observed approximately 7 times higher CTB binding capacity on the fucosyl-GM_1_ surface than the GM_1_ surface. This was unexpected as GM_1_ is known to exhibit higher binding avidity (i.e. lower dissociation constant). Moreover, we observed the very weak binding receptor, GM_2_, was activated by fucosyl-GM_1._ This activation increased the number of CTB molecules binding to the fucosyl-GM_1_-GM_2_ lipid bilayer. To the best of our knowledge, these phenomena have never been reported. To explore the observed phenomena theoretically, we analyzed the stepwise binding model reported by Klassen and his coworkers [5]. From probing Klassen’s model, we found binding cooperativity plays an essential role in CTB binding. This analysis may answer the question posed by Yanagisawa et al., “why is the amount of bound CTB not correlated with GM_1_ expression level in neural cells?” [9]. Our discovery demonstrates the essential nature of cooperativity in multivalent GBP binding. Furthermore, our sensor provides a facile method to analyze GBP cooperativity as its fluidic bilayer provides unconstrained binding for CTB and its high-throughput methodology enables the study of cross-reactivity. By leveraging our nanocube-based lipid bilayer array, we can assist biologists in dissecting the complex binding mechanisms of multivalent GBPs.

## Materials and Methods

### Materials

Monosialogangliosides, GM_1,_ (2Galβ1-3GalNAcβ1-4(Neu5Acα2–3)Galβ1-4Glc-Ceramide) were acquired from three different vendors, including Matreya LLC (State College, PA), Avanti Polar Lipids (Alabaster, AL), and Sigma-Aldrich. Fucosylated monosialoganglioside GM_1_ (Fucα1-2Galβ1-3GalNAcβ1-4(Neu5Acα2–3)Galβ1-4Glc-Ceramide, fucosyl-GM_1_), was purchased from Matreya LLC. 1, 2-dioleoyl-sn-glycero-3-phosphocholine (DOPC), 1, 2-dioleoyl-sn-glycero-3-phospho-L-serine—sodium salt (DOPS), and 1,2-dioleoyl-sn-glycero-3-phosphoethanolamine-N-(biotinyl) (biotin-PE) were obtained from Avanti Polar Lipids (Alabaster, AL). Cholera Toxin B subunit (CTB) from *Vibrio cholerae*, GM_2_ (3GalNAcβ1-4(Neu5Acα2–3)Galβ1-4Glc-Ceramide), streptavidin from *Streptomyces avidinii* (StP), copper (II) chloride dihydrate, polyvinylpyrrolidone (PVP) (MW ~55,000), tetraethyl orthosilicate (TEOS), and silicone oil (useable range -50°C to +200°C) were purchased from Sigma-Aldrich. Silver nitrate (Premion, 99.9995%) was purchased from Alfa-Aesar. 1,5-Pentanediol 98% (PD) was purchased from Acros Organics through Fisher Scientific. The calibration experiments were performed in phosphate buffered saline 1X phosphate buffered saline (PBS) diluted from a 10X PBS stock from CulGenX. CTB binding was performed in 1X Tris-buffered saline (TBS) (20mM Tris 0.9% NaCl pH~7.4) diluted from a 10X TBS stock from Sigma-Aldrich.

### Methods

#### Silver Nanocube Synthesis Procedure

The nanocube synthesis procedure was taken from Tao et al. [20]. The procedure was based on the polyol method and described in brief as follows. First, 0.2 g of PVP was dissolved into 10 mL of PD. Next, 0.2 g of AgNO_3_ was dissolved into 10 mL of PD with 30 μL of a 0.082 g/mL CuCl_2_ in PD solution. Then, 20 mL of PD was heated in a 190°C silicon oil bath. After the PD was heated sufficiently, 500 μL of AgNO_3_ solution and 500 μL of the PVP solution were added sequentially every minute. This was continued until all 10 mL of both the AgNO_3_ and PVP solutions were added. When finished, the nanocubes were washed with 200 proof ethanol using a centrifuge.

#### Modified nanocube silica coating procedure

Our silica coating procedure was adapted from Wu et al. with few alterations and presented with alterations as follows [19]. To improve silica shell quality in the scaled-up synthesis batch, the silica coating reaction was conducted in 2-propanol, instead of ethanol. 20 mL of stock silver nanocube stored in ethanol was first transferred into 2-propanol. Then, the silver nanocube solution was suspended into 55 mL of 2-propanol and mixed with 22.1 mL of water, 6.80 mL of TEOS, and 3.4 mL of 0.84% ammonium hydroxide solution. Next, the solution was stirred at room temperature for 80 minutes. After the reaction finished, 50 mL of ethanol were added to quench the reaction. The resultant particles were washed with Milli-Q^®^ water a few times, and stored in Milli-Q^®^ water for future use. (Fig 1b)

#### Calibration of the silica coated silver nanocubes

The silica thickness of silica coated silver nanocube was imaged directly by transmission electron microscope (FEI Technai G2 F20 FE-TEM). The size and uniformity of the silver nanocubes prior to silica coating was determined by direct imaging with a scanning electron microscope (FEI Quanta 600 FE-SEM). The sensitivity of the silica coated silver nanocubes was determined by the method given by Wu et al. using a figure of merit (FOM) that was calculated by dividing refractive index sensitivity by the line width of the resonance spectrum [19].

The relationship between the quadrupole LSPR peak shift and the surface mass density of protein bound was measured by binding streptavidin to biotin-PE on the bilayer surface [19] (S2 Fig). Our previous work established a protocol to change bound streptavidin by titrating streptavidin concentration [19]. Briefly, bilayer (89% DOPC/10% DOPS/1% biotin-PE) coated Ag@SiO_2_ nanocubes were titrated with streptavidin in a 384 well plate (Greiner Bio-one). The average streptavidin surface density on nanocubes was evaluated by approximating each lipid as a single DOPC lipid to obtain the surface area coverage in the supported bilayers [19].

#### Supported Bilayer Preparation

Small unilamellar vesicles (SUVs) were prepared as follows. The desired composition of lipids in chloroform was mixed and then dried using a rotary evaporator (Heidolph Hei-VAP Value^®^). Then, the dried lipids were rehydrated with Milli-Q^®^ water and extruded through 100 nm polycarbonate filters (Whatman) using a Mini-extruder (Avanti Polar Lipids) to achieve an extruded lipid concentration of 3 mg/mL. Supported lipid bilayers were formed by a modified vesicle-fusion technique. In this technique, Ag@SiO_2_ nanocubes were sequentially added into a SUV solution with a high SUV concentration in the initial coating solution. Briefly, 10 μL of nanocube solution and 30 μL of 2X TBS buffer were added to 20 μL of concentrated SUV solution (3 mg/mL) followed by 10 seconds of sonication in a bath sonicator (Branson). Then, 10 μL of nanocube solution and 10 μL of 2X TBS buffer were added followed by 10 seconds of sonication. This process was repeated until all of the nanocube solution had been added. After coating the supported bilayer, TBS buffer was added to the solution to reach the desired concentration of salt (1X TBS), SUV’s, and nanocubes in the final solution.

#### Protein Binding Measurement

Bilayer coated nanocubes were incubated with the desired protein concentration in a 384 well plate for 1.5 hours. Blank solutions were prepared for each CTB concentration by mixing buffer, SUVs, and CTB corresponding to that composition. Next, the 384 well plate was placed in a vacuum chamber at 40 cm Hg of vacuum for 15 minutes to remove air bubbles before collecting extinction spectra with a UV/Vis microplate spectrophotometer equipped with a CCD (FLUOstar Omega^®^, BMG-Labtech). The location of the quadrupole LSPR peak was detected by fitting a seventh order polynomial to the spectrum. The fitted spectrum resulted from averaging 200 flashes per well at a 1 nm spectral resolution; the scanning rate for each well was less than 1 second. All experiments were performed at room temperature.

The total amount of the CTB was calculated from the amount of CTB added. The amount of bound CTB was calculated from the observed LSPR shifts. The individual replicate LSPR shift was obtained by finding the wavelength corresponding to the maximum optical density given by the seventh order polynomial peak fitting. Then the LSPR shifts of eight replicate wells were averaged to give the observed LSPR shift used to calculate the amount of bound CTB based on the Streptavidin-Biotin binding calibration. The difference between the total amount of CTB and the amount of bound CTB gave the amount of unbound CTB.

#### Cryo-TEM Measurements

Supported lipid bilayer morphology and quality was assessed by cryo-TEM (FEI Technai G2 F20 FE-TEM with a Gatan Tridiem^®^ GIF-CCD using a Gatan 626 cryo-specimen holder) (Fig 1c). Measurements were conducted on silica-coated silver nanoparticles supporting 88%-90% DOPC/10%DOPS/0-2% ganglioside lipid bilayers. The lipid bilayers were coated 1 hour before vitrification and stored in a 1X TBS buffer. The samples were vitrified on Quantifoil^®^ grids, holey carbon films (shape R2/2); the sample, suspended in 1X TBS aqueous solution, was rapidly frozen via submersion in liquid ethane and cooled to liquid nitrogen temperature using an FEI Vitrobot^®^.

#### Simulation of Localized Surface Plasmon Resonance (LSPR)

To simulate the electric field environment near a single silica-coated silver nanocube, the observed particle geometry (from TEM) was used to construct a Finite Element Method (FEM) model in the COMSOL Multiphysics RF Module^®^. We simulated a quarter cube of side length 112 nm, radius of curvature 12 nm, and silica shell thickness of 4 nm on the sides smoothly transitioning to 3.1 nm on the corners, oriented such that symmetry was imposed in the planes perpendicular to the x and y axes. A plane wave, propagating in the +x direction and polarized along the z axis, was introduced and then Maxwell’s equations were solved for the resulting scattered electric field. The extinction coefficient, which is equal to the extinction spectrum when scaled by path length and particle concentration, was calculated from the scattered field. The simulation was repeated, varying dielectric properties of the model environment, until the refractive index sensitivity calibration experiment was repeated *in silico*.

#### Statistical Analysis and Regression

Each data point of each binding curve is represented as the mean ± standard deviation (S.D.) where n = 8. Then, Hill-Waud model was fit to the binding curves. To fit the Hill-Waud model to our data, we used the Levenberg Marquardt iterative algorithm (*fitnlm* function in Matlab 2013b^®^). The choice of the Levenberg Marquardt function was based on fitting a relatively simple function, desiring fast solution times, and having very precise instrument measurements of the wavelength. The *fitnlm* function returned the calculated value, standard error, and R^2^ value presented for each variable in Table 1.

**Table 1:** Hill-Waud Equation Fitting Parameters for various ganglioside compositions.

Lipid composition (mol %)					Hill's Equation fitting parameters			
DOPC	DOPS	GM_1_	fucosyl-GM_1_	GM_2_	*K*_*h*_±S.E. of the estimate (n = 19) (nM)	*C*_*max*_ ±S.E. of the estimate (n = 19) (nM)	*n* ±S.E. of the estimate(n = 19)	R^2^
89	10	1	0	0	5.6 ± 0.6	5.3 ± 0.1	2.25 ± 0.45	0.943
88	10	2	0	0	14.5 ± 1.0	11.5 ± 0.3	1.93 ± 0.25	0.968
86	10	4	0	0	48.0 ± 3.0	41.0 ± 1.8	2.79 ± 0.45	0.959
80	10	10	0	0	151.0 ± 7.0	79.0 ± 2.3	2.79 ± 0.31	0.986
89.5	10	0	0.5	0	59.4 ± 5.7	12.0 ± 0.3	0.78 ± 0.05	0.993
89	10	0	1	0	270.8 ± 56.8	32.5 ± 1.9	0.69 ± 0.06	0.992
88.5	10	0	1.5	0	129.1 ± 13.0	34.4 ± 1.1	1.06 ± 0.09	0.989
88	10	0	2	0	251.8 ± 47.1	83.5 ± 5.3	0.89 ± 0.10	0.985
88	10	0	0	2	(1.661±n/a) ∙10^8^*	(6.39±2.91) ∙10^3^*	0.70±0.04	0.971*
88	10	0	0.5	1.5	563.4 ± 156.4	88.7 ± 8.5	0.85 ± 0.11	0.982
88	10	0	0.75	1.25	380.4 ± 159.1	91.0 ± 9.1	0.56 ± 0.07	0.978
88	10	0	1	1	830.5 ±114.6	96.6 ±4.9	0.82 ±0.04	0.998
88	10	0	1.5	0.5	682.7 ± 190.5	92.6 ± 7.9	0.69 ± 0.05	0.993

* The value ± S.E. of the estimate given is highly uncertain.

## Results

### Scaled-up synthesis of Ag@SiO_2_ nanocube for high-throughput detection

Analysis of multivalent ganglioside-CTB interactions required many measurements at different experimental conditions with replication. Therefore, large-scale synthesis of high quality sensors was critical to perform the complex analysis. The current silver nanocube synthesis reached mass production (allowing up to 20,000 measurements per batch); however, the silica coating process was still at a smaller scale (<5mL batch reactor). The prior protocol required extensive sonication during the entire reaction. However, it was difficult to provide sufficient mixing power in a large batch reactor by sonication. To scale up Ag@SiO_2_ synthesis, we modified the prior Stöber silica coating procedures [19]. Instead of ethanol, 2-propanol was used in silica coating reaction to increase the hydrolysis reaction rate of TEOS [21]. This was necessary as ammonium hydroxide forms an ammonium silver complex that prevents the formation of SiO_2_ shell [22]. Hence, the hydrolysis reaction rate was increased to be competitive with the formation of the ammonium silver complex and improve SiO_2_ shell uniformity. In addition to preventing SiO_2_ shell formation, the ammonium silver complex occurs preferentially at the exposed {111} crystal plane resulting in etching. The localization of the ammonium silver complex formation is due to the strong adsorption of PVP on {100} facets that prevents/slows the reaction between silver and ammonium ions [23]. Problematically, the etching by the ammonium silver complex results in rounded nanocube corners, reducing electro-magnetic field enhancement and lowering sensor sensitivity. Thus, the increased hydrolysis rate minimized these drawbacks and improved the quality of silica coating in scaled-up synthesis (S1 Fig).

**Fig 2:**
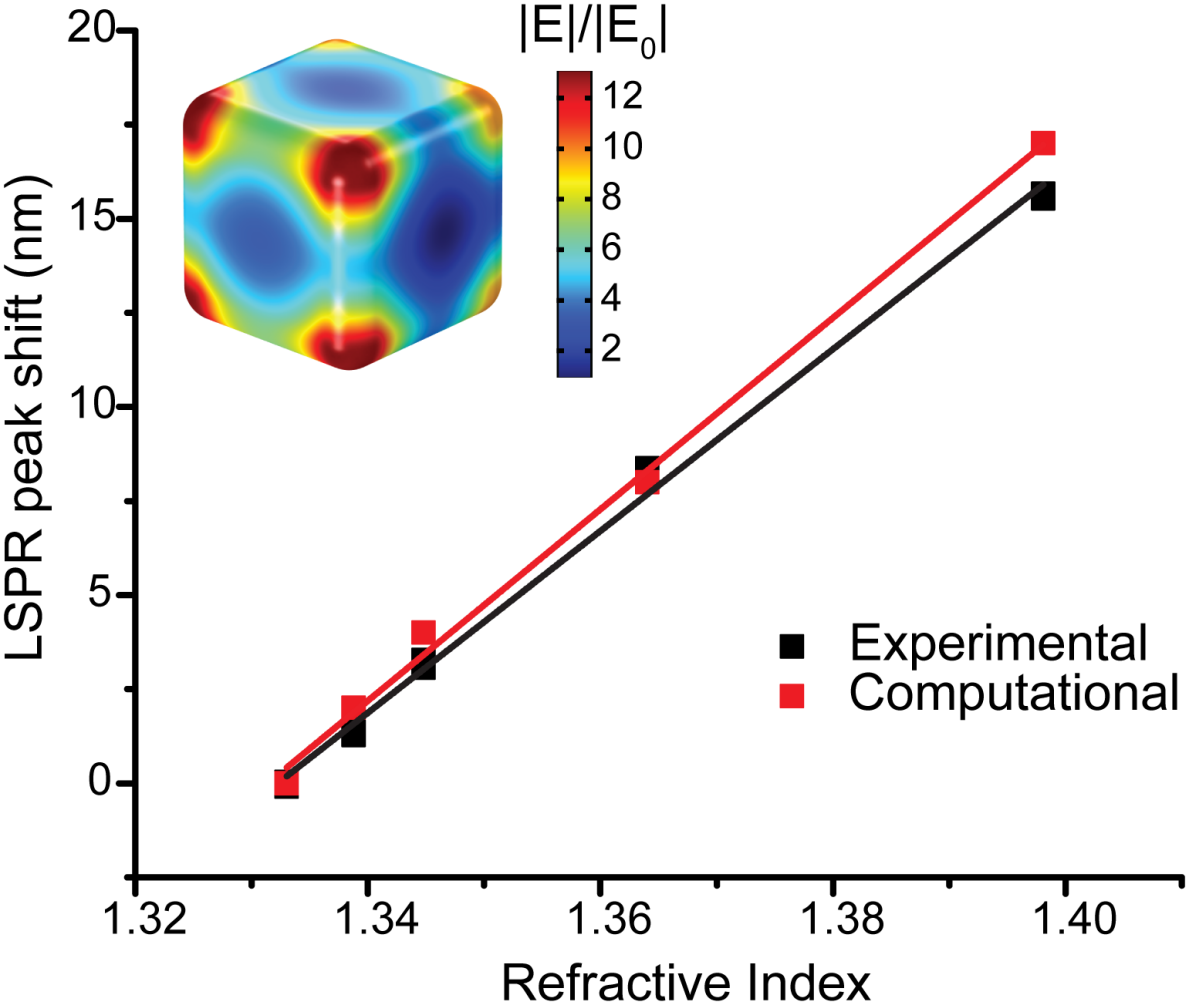
High quality nanocube synthesis and sensor sensitivity confirmed with single particle computational modeling. Refractive Index (RI) vs. change in quadrupole LSPR peak location using a single Ag@SiO_2_ nanocube simulated in COMSOL Multiphysics and using Ag@SiO_2_ nanocubes in various glycerol-water mixtures measured with a spectrophotometer. The experimental slope is 242 nm/RI unit ±4 (S.E. of the estimate n = 20). The computational slope is 254 nm/RI ± 9 (S.E., n = 5). The details are in the Methods Section. (Inset) A computational model of a single Ag@SiO_2_ nanocube that was simulated in COMSOL Multiphysics^®^ using the Radio Frequency module.

The quality of Ag@SiO_2_ nanocube was determined by TEM (Fig 1b and S1 Fig). The refractive index sensitivity of Ag@SiO_2_ sensor was measured by suspending the sensor in various glycerol-water solutions (Fig 2). In order to confirm the variation of Ag@SiO_2_ nanocube, we modeled the LSPR of Ag@SiO_2_ nanocubes based on their geometry observed in TEM image (averaged length 112 nm and corner curvature 12 nm). In Fig 2, the experimental sensitivity has been compared with the LSPR simulation. The refractive index sensitivity of the single simulated cube (254±9 nm/RI, S.E., n = 5) was observed to be very similar to the ensemble average of experimental response (242±4 nm/RI, S.E., n = 20). This simulation result suggested that our nanocube synthesis yielded high quality nanoparticles with low polydispersity.

After characterization of Ag@SiO_2_, the cubes were covered with a supported lipid bilayer and characterized again. Supported lipid bilayer morphology and quality was imaged by cryo-TEM. (Fig 1c) The Ag@SiO_2_ nanocube was uniformly covered by a continuous supported lipid bilayer with approximately 4nm thickness, which is similar to the known thickness of a lipid bilayer [24].

### Multivalent binding between CTB and GM_1_ ganglioside

Cholera toxin subunit B (CTB) binding to the lipid bilayer was measured by observing the shift of the quadrupole LSPR scattering peak of our nanocube based sensor. All measurements were conducted with eight replicates for each protein concentration in a 384 well plate using high-throughput microplate reader. We followed the protocol reported by Wu et al. to calibrate the correlation between quadrupole LSPR shift and protein density by titrating bound streptavidin onto the lipid bilayer containing biotin-PE (S2 Fig) [19]. This correlation allowed quantification of an absolute bound CTB density on ganglioside presenting surfaces. Control experiments were also carried out on the lipid bilayer with 90% DOPC and 10% DOPS.

To measure the cooperativity of multivalent CTB binding, a classic multivalent binding model, the Hill-Waud binding model (Eq 1), was used to fit the equilibrium binding curves [25].

$$C=\frac{C_{max}{\left[P\right]}^{n}}{K_{h}^{n}+{\left[P\right]}^{n}}$$ (1)

*C* is the concentration of bound CTB to the cell membrane surface and [*P*] is the concentration of unbound CTB in the solution. The fitted parameters are: *C*_*max*_, the maximum binding capacity of membrane surface; *K*_*h*_, the apparent dissociation constant; and *n*, the Hill coefficient of cooperativity. If there was no cooperativity between two bound gangliosides, *n* was equal to one. When *n* was larger or smaller than one, it represented positive or negative cooperativity, respectively.

**Fig 3:**
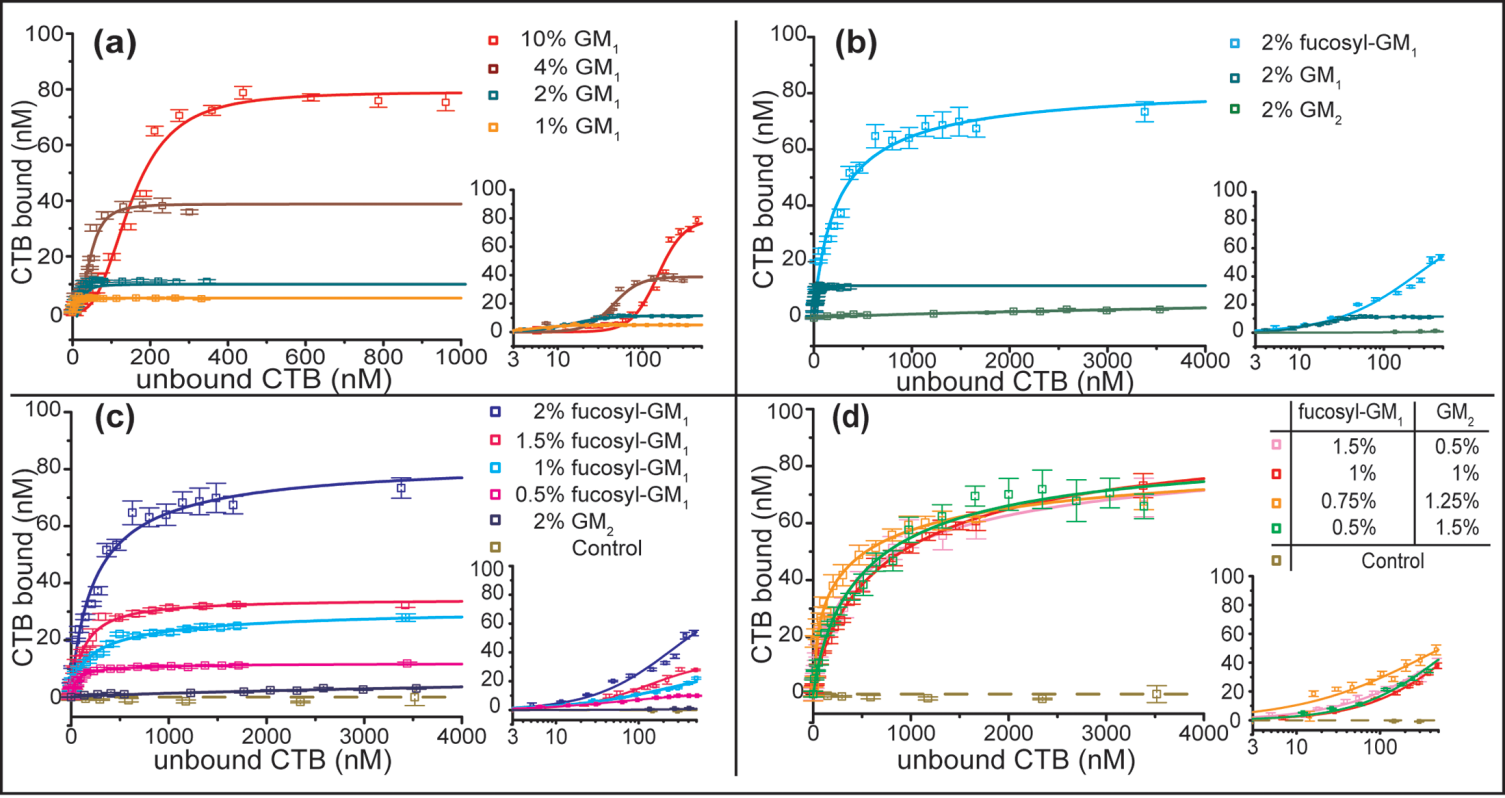
Equilibrium binding data for CTB binding with different gangliosides. The insets represent the same binding curves on semi-log scale to better show the data points at low concentrations. (a) CTB-GM_1_ binding data at differing surface densities. (b) Homogeneous receptor CTB-(GM_1_-like) ganglioside binding data with constant surface density. (c) CTB-fucosyl-GM_1_ & CTB-GM_2_ binding data at differing surface densities. (d) Heterogeneous CTB-ganglioside mixture binding data at constant surface density. A control of 90% DOPC/ 10% DOPS was used to verify the absence of non-specific binding. Data points are reported as mean ± S.D (n = 8).

In the GM_1_ binding experiments, the nanocube sensors were coated with lipid bilayers containing various surface densities of GM_1_ (1, 2, 4, and 10 mol%) separately for CTB binding. The binding curves were measured by titrating CTB in separate wells of a 384 well plate, with 8 replicates per titration, at each mol% GM_1_ (Fig 3a). The CTB-GM_1_ binding system provided a good comparison for our nanocube sensor with other established methods because its binding mechanism has been well studied [4, 5, 12, 25–29]. The fitted parameters of the Hill-Waud equation are shown in Table 1. Intuitively, increasing GM_1_ density increased the binding capacity (*C*_*max*_) of the bilayer surfaces. We also observed that the *K*_*h*_ of CTB-GM_1_ binding increased with increasing GM_1_ mol%. Furthermore, Cremer and his coworkers observed the same phenomena on supported lipid bilayers using fluorescent microscopy [25, 26]. They suggested the clustering effect of gangliosides in supported lipid bilayers at higher surface densities inhibits CTB binding [25]. In addition, the positive cooperativity of CTB-GM_1_ binding was observed from the fitted Hill’s coefficients (*n*), and the measured coefficients were similar to the values reported in literature [25, 26].

### Multivalent binding between CTB and GM_1_-like gangliosides

Beyond GM_1_, other GM_1_-like gangliosides associated with CTB have been identified. Most of the previous studies identified GM_1_- and GM_1_-like ganglioside-CTB binding avidities with isothermal titration calorimetry (ITC), mass spectrometry (MS), or immobilized receptors on solid substrates [2, 4, 5, 27, 28, 30]. Some studies conducted the CTB-ganglioside binding measurements using fluidic lipid bilayers [12, 15, 25, 26, 29, 31–34]. Regardless of measurement technique, these studies often reported the apparent association constants and thermodynamic parameters of CTB binding to various gangliosides, but few of them analyzed the cooperative actions between bound gangliosides. To further investigate cooperative interaction in multivalent CTB binding to GM_1_-like gangliosides, we selected two gangliosides, fucosyl-GM_1_ and GM_2_, which exhibit mild and weak binding avidity to CTB, respectively.

Masserini et al. [34] and Iwabuchi et al. [35] found the binding association constants of CTB with fucosyl-GM_1_ or GM_1_ ganglioside were comparable. Several studies reported diverse association binding constants of CTB with GM_2_, but the binding avidity of GM_2_ was generally much lower (10~10^5^ times weaker) than GM_1_ [2, 27, 28, 30]. To investigate the multivalent binding of these two gangliosides, we measured the binding curves at 2 mol% surface density of each ganglioside and fitted the Hill-Waud equation to the curves (Fig 3b and Table 1). The *K*_*h*_ of fucosyl-GM_1_ was approximately one order of magnitude higher than GM_1_. A very weak binding of CTB to GM_2_ was observed. We did not reach the plateau region for GM_2_ binding curve, as the concentration of CTB was far beyond physiologically relevant conditions [36]. Regardless, this low binding avidity was not surprising because Lauer et al. and MacKenzie et al. could not detect any binding of CTB to fluidic bilayer surfaces with GM_2_ receptors [12, 29].

We observed the Hill coefficient is almost always less than one for fucosyl-GM_1_, even when accounting for the standard error of the estimate. This indicated that the interaction between bound fucosyl-GM_1_ and its free counterpart is negatively cooperative. This implies the initially bound receptor lowers the binding avidity for future binding events. The fitted parameters, *K*_*h*_ and *C*_*max*_, of the GM_2_ binding curve were not very accurate as these two parameters depend highly on the plateau region of binding curves. The lower CTB concentration range mainly determined the fitting of *n* value; hence, the negative cooperativity of GM_2_ binding was still convincing (*n* = 0.70±0.04, S.E., n = 19). To the best of our knowledge, such negative cooperativity of CTB with GM_1_-like gangliosides has not yet been reported.

The most surprising observation was that the binding capacity (*C*_*max*_) of fucosyl-GM_1_ was more than 7 times greater than GM_1_ at the same surface density (2 mol%). This seemingly contrasted with the dissociation constant of GM_1_, which was more than 16 times lower. To exclude the potential experimental error from the degradation of GM_1_ reagents, we performed the same binding measurements with GM_1_ gangliosides acquired from three different vendors (Sigma-Aldrich, Avanti, and Matreya LLC). The binding curves of the three GM_1_ gangliosides across eight replicates were very consistent (S3 Fig). These experiments demonstrated that the degradation of GM_1_ reagent was not the cause of the lower binding capacity of the CTB- GM_1_ binding system. These data sets also indicated that the experimental variation of our binding measurements was low. The GM_1_ used for all the other experiments was obtained from Matreya LLC.

### The influence of Cooperativity on binding capacity

To the best of our knowledge, the unusually high binding capacity observed for fucosyl-GM_1_ has not been reported. Based on the fitting of the Hill-Waud model, we found that the major difference between GM_1_ and fucosyl-GM_1_ was the Hill coefficient of cooperativity, n. We suspected the reduced cooperativity of fucosyl-GM_1_ binding led to the higher observed binding capacity. To understand the binding mechanism, we explored a stepwise binding mechanism of CTB. Klassen and his coworkers used direct electrospray ionization mass spectrometry (ESI-MS) to investigate the stepwise binding of GM_1_ to CTB [5]. They established a comprehensive stepwise binding model (S4 Fig) and determined the apparent association constants for three different states (*K*_*1*_, *K*_*2*_, *K*_*3*_), including zero, one, or two receptor-bound nearest neighbors [5]. The binding affinity was enhanced by a factor of approximately two when there was a bound GM_1_ next to the binding pockets. This comprehensive, single receptor binding model allowed us to explore the detailed binding mechanism by calculating the concentration of each binding state.

**Fig 4:**
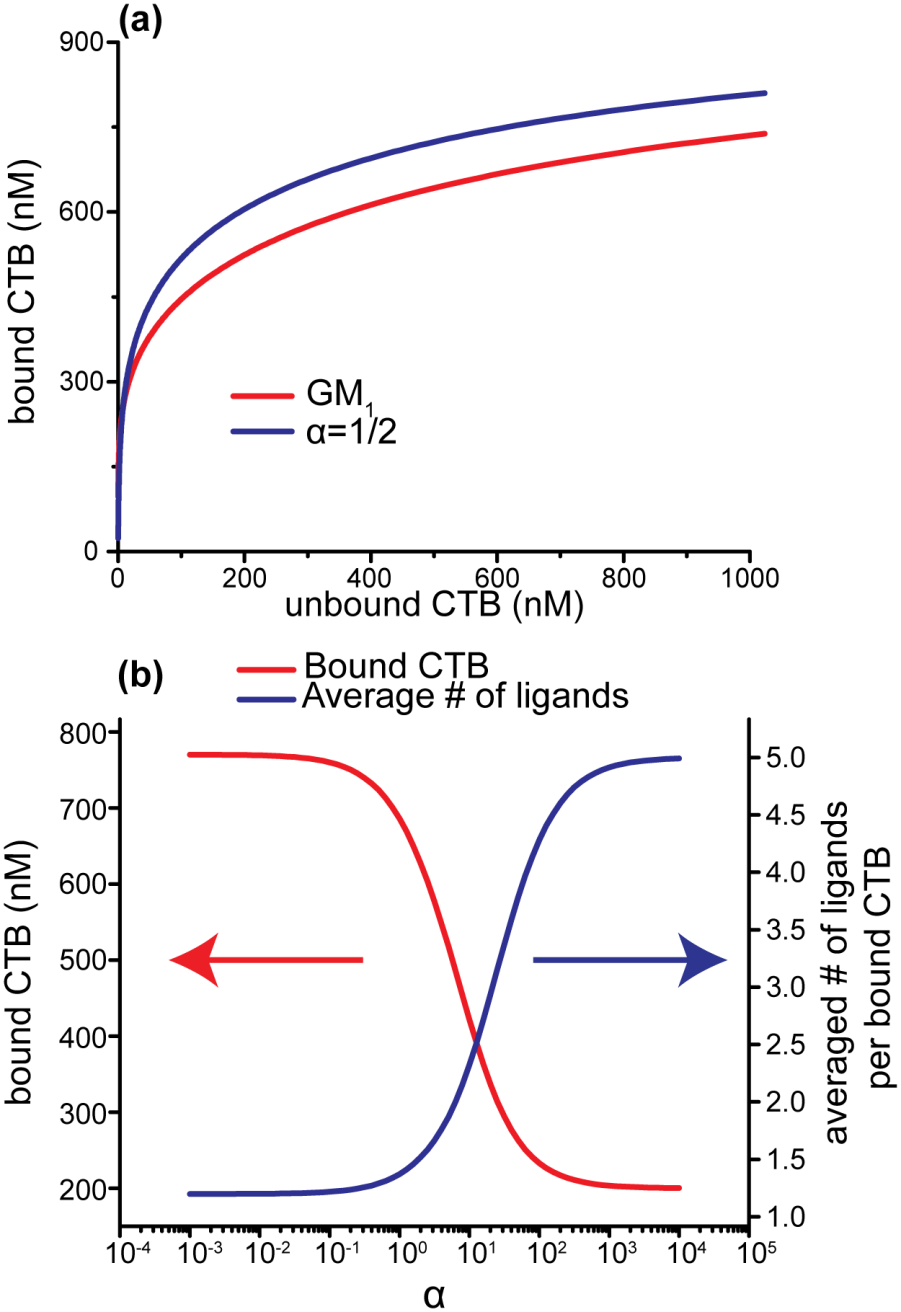
Simulated binding with varying cooperativity based on Klassen’s theoretical model. (a) Total bound CTB as a function of unbound CTB for varying cooperativity ratios. The negative cooperativity (α = ½) could reach a higher binding capacity than GM_1_ (α ~ 2) (b) Bound CTB and average number of ganglioside receptor per bound CTB molecule as a function of the affinity scaling factor, α, with an unbound CTB concentration of 500 nM.

We modeled Klassen’s stepwise binding and calculated the concentration of each CTB-GM_1_ bound state at different CTB concentrations. The equation we adapted from Klassen’s model is summarized in S1 File. The association constants of fucosyl-GM_1_ for each individual state were not measured, so we added a factor ‘α’ to estimate, from initial binding (*K*_*1*_), the affinity of the CTB binding subunit when there are one (*K*_*2*_) or two (*K*_*3*_) bound gangliosides in the nearest neighbors. α > 1 represented positively cooperative binding and α < 1 represented negatively cooperative binding. Using the empirical values obtained by Klassen et al., α ≈ 2 for GM_1_, indicating positive cooperativity. This theoretical model demonstrated that receptors with reduced cooperativity compared with GM_1_ (α < 2) could reach a higher binding capacity than GM_1_ (α~2), despite having the same initial binding affinity (*K*_*1*_) (Fig 4a). Since the overall *K*_*h*_ was higher for fucosyl-GM_1_ (Table 1), it was reasonable to conclude that *K*_*1*_ of fucosyl-GM_1_ would be less than the *K*_*1*_ for GM_1_. To reflect this qualitatively, we changed the value of *K*_*1*_ to half of its original value to see if that altered our observation (S5 Fig). Despite this change, we observed that the binding capacity continued to be higher for receptors with lower cooperativity. To understand this unusual behavior, we calculated the concentration of CTB in each bound CTB state (S6 Fig). We found the model predicted that the number of CTB molecules binding with two or more gangliosides was higher when the binding cooperativity was positive. For positive cooperativity, the bound gangliosides enhanced the binding affinity of unbound binding subunits, making the second and higher order binding events more favorable. In contrast, the average number of gangliosides per CTB was closer to one when the binding was negatively cooperative. As shown in plotted model data in Fig 4b, the average number of bound ganglioside receptors per CTB increased when cooperativity increased. This meant that the model predicted a single CTB bound more gangliosides and reduced the number of free gangliosides available on the lipid bilayer during positively cooperative binding. Therefore, the total binding capacity of positive cooperative binding was lower. Recently, Klassen and his coworkers reported a similar phenomenon using nanodiscs-ELI-MS technology [15]. They found that a majority of CTBs bound to only one ganglioside when three gangliosides, GM_1_, GM_2_, and GM_3_, were incorporated in separate nanodiscs. If three gangliosides were mixed in a single nanodisc, most CTBs bound to two gangliosides. We believe the reduced cooperativity, relative to GM_1_, of GM_1_-like gangliosides led to their experimental findings. Our theoretical analysis of the stepwise binding model has demonstrated that cooperativity significantly influences binding capacity. Thus, the unusually high binding capacity of fucosyl-GM_1_ might be attributed to its lower cooperativity.

### The influence of mixed gangliosides on multivalent binding

After demonstrating that cooperative interaction between molecules of a single ganglioside type significantly influences multivalent binding, we hypothesized that cooperative interaction between heterogeneous gangliosides might also play an essential role in CTB binding mechanisms. In order to test this hypothesis, we mixed weak and mild binding gangliosides, GM_2_ and fucosyl-GM_1_, at different surface densities to investigate their cooperative interactions. The total surface density of GM_2_ and fucosyl-GM_1_ was fixed at 2% and we varied the ratio of these two gangliosides (0.5%/1.5%, 0.75%/1.25%, 1%/1%, and 1.5%/0.5% of GM_2_/fucosyl-GM_1_). For comparison, we also measured the binding curve of individual fucosyl-GM_1_ at 0.5, 1.0, 1.5, and 2.0 mol% surface density. The binding curves and the fitted Hill-Waud parameters were recorded in Fig 3b and Table 1. It is important to recognize that the *K*_*h*_ and *n* values do not carry the same physical meaning when commuted to the two-component binding model. This is because the Hill-Waud model was derived from single-receptor system (hemoglobin-oxygen binding). This means that a two-component fitted Hill-Waud model is an empirical model. Thus, *K*_*h*_ and *n* values must be considered apparent terms representing the combined effects of the two components; this limits any conclusions that could be drawn from a thermodynamic analysis of the fitted model. Due to these limitations, we do not refer to *C*_*max*_ when referencing the two-component system, but rather refer to the binding capacity observed at the highest tested concentration, 3.4 μM unbound CTB (*C*_*3*.*4μM*_). By this measure, we can draw direct comparisons to the pure component systems.

As expected, without GM_2_, reducing fucosyl-GM_1_ surface density on the membrane surface reduced the maximum binding capacity (*C*_*max*_). The dissociation constant also decreased at lower fucosyl-GM_1_ surface density. The same trend was observed in our GM_1_ measurements and the GM_1_ measurements reported by Cremer and his coworkers [25]. Interestingly, the additional GM_2_ gangliosides compensated for the loss of fucosyl-GM_1_ (for the tested conditions) and the highest observed CTB binding (*C*_*3*.*4μM*_) for GM_2_/fucosyl-GM_1_ mixtures reached values similar to *C*_*3*.*4μM*_ for 2 mol% fucosyl-GM_1_. If CTB bindings to GM_2_ and fucosyl-GM_1_ were independent, the *C*_*3*.*4μM*_ for GM_2_/fucosyl-GM_1_ mixtures should equal the sum of the *C*_*3*.*4μM*_ values for equivalent single-receptor bilayers containing GM_2_ or fucosyl-GM_1_. This is not the case. For instance, the total bound CTB at 3.4μM (*C*_*3*.*4μM*_) on 1.5% GM_2_/ 0.5% fucosyl-GM_1_ surface is approximately 7 times higher than the summation of pure component systems. These data sets suggested the multivalent binding depends on the complex pattern of gangliosides.

## Discussion

We performed direct measurements to demonstrate the essential nature of binding cooperativity in pentavalent CTB binding to gangliosides on lipid bilayer surfaces. Our stepwise reaction analysis confirmed the higher binding capacity of fucosyl-GM_1_ compared to GM_1_, and suggested that this might be induced by the reduced binding cooperativity of fucosyl-GM_1_. The observed binding capacity (*C*_*3*.*4μM*_) of the membrane with the GM_2_/fucosyl-GM_1_ mixture also markedly increased compared to the summed total binding capacity of equivalent membranes with a single type of ganglioside. This change may indicate a conformational change induced by either fucosyl-GM_1_ or GM_2_ to alter binding preferences and/or inter-subunit distances. The other possible explanation is the reduction in membrane fluidity, which can improve CTB binding as observed by Terrell et al. [33]. However, this seems less likely than cooperative interactions between fucosyl-GM_1_ and GM_2_ because the total ganglioside surface density was maintained at 2% and both gangliosides have very similar molecular structures. Thus while we cannot identify the exact mechanism at this point, we are reasonably certain that cooperativity between fucosyl-GM_1_ and GM_2_ can significantly enhance the bound CTB concentration.

Biologists have also observed such unexpected binding between CTB and mixed gangliosides [9, 37, 38]. In the past, CTB has been used to quantify the amount of GM_1_ that was present in a cell membrane [38, 39], but the validity of this approach was refuted by Yanagisawa et al [9]. In the absence of GM_1_, Yanagisawa et al. observed strong reactivity between CTB and embryonic neuroepithelial cells and attributed this phenomenon to the expression of GM_1_- like ganglioside [9]. More recently, some studies have used local CTB concentration differences as a means to identify and/or quantify lipid rafts [40, 41]. However, it was recommended that this approach be combined with other methods before asserting the presence of lipid rafts based on CTB binding to multiple gangliosides [35, 38]. Thus, for CTB to be used in quantification and/or identification of GM_1_, the analysis must be combined with another analysis method, such as MS/MS or ITC, or a tool must be used that can differentiate between the various CTB-ganglioside bindings and account for cooperativity.

Similar cooperativity may appear in the other multivalent GBPs (e.g. lectins). Lectins, often consisting of multiple identical binding subunits, are widely used in glycome analysis (e.g. lectin microarray or cell staining) [42–45]. From conventional glycoarray analysis, it is known that some lectins preferentially bind to the same glycan structure; however, their binding specificities to a heterogeneous cell surface are very different. For instance, Galβ-GalNAc is the preferred glycan binding structure for *Amaranthus caudatus* lectin, *Agaricus bisporus* lectin, *Colchicum autumnale* lectin, *Maackia Amurensis* lectin I, and *Phytolacca americana* lectin, but these lectins exhibited varying binding specificities to different types of cells [43]. We hypothesize the cooperative efforts among heterogeneous glycans may contribute to lectin binding specificities. Hence, in order to understand the hetero-multivalent binding, a proper analytical tool is of critical importance.

Cooperative binding between heterogeneous glycan structures is difficult to observe by conventional glycan microarray analysis because glycan microarrays often detect only the interaction with isolated glycans. Although some studies have printed mixed glycans on solid substrates [46, 47], immobilized glycans cannot completely achieve multivalent binding with proteins. Therefore, there is a need for our nanocube-based sensor with gangliosides inserted into a fluidic supported lipid bilayer that enables the detection of hetero-multivalent binding. Combined with glycolipid synthesis, such as the neoglycolipid (NGL) technology [48], the nanocube-based lipid bilayer array can be an attractive tool for studying GBP-glycan recognition.

Our sensing platform greatly improves on traditional methods by taking advantage of supported lipid bilayer technology. Our platform is also an improvement on current fluidic bilayer methods in several ways. First, our nanocube-based sensor is inexpensive. Second, our assay is compatible with high-throughput analysis methods, allowing the thorough study of complex binding systems. Third, our nanocube-based lipid bilayer array is compatible with common laboratory equipment, enabling its widespread use while still maintaining sensitivity.

## Conclusion

Using our nanocube-based system, we were able to experimentally elucidate the relationship between cooperativity and maximum CTB-ganglioside binding and the effects of mixing multiple recognized gangliosides in a single lipid bilayer system. Through experimental measurements and representative stepwise binding analysis, we demonstrated that binding cooperativity is essential in multivalent CTB binding. The attenuation or enhancement of CTB binding was shown not to simply be controlled by any one of the gangliosides; the reactivity depended on cooperative interactions within the entire ganglioside complex. This analysis required many replications and individual experimental conditions to investigate, despite only analyzing a single two component cross-reactivity test. We were able to obtain all of this data with replication because of our highly accessible and high-throughput nanocube-based lipid bilayer array that can be leveraged by biological communities to dissect additional complex binding models of multivalent binding proteins.

## Supporting Information

S1 Fig TEM image comparison of silica coating procedures. (a) A TEM image of the silica shell coated onto Ag nanocubes in 2-propanol. (b) A TEM image of the silica shell coated onto Ag nanocubes in ethanol.
(PDF)

S2 Fig Equilibrium StP- Biotin binding for sensor calibration. Streptavidin (StP)-biotin-DPPE binding data assuming that all StP is bound to a biotin group resulting in the observed LSPR shift. Data are reported as mean ± S.D. (n = 8).
(PDF)

S3 Fig GM_1_ quality comparison across vendors. Comparison of GM_1_ gangliosides obtained from various companies with fucosyl-GM_1_ plotted for reference. Data are reported as mean ± S.D. (n = 8).
(PDF)

S4 Fig Stepwise CTB binding model with a single type of ganglioside. The stepwise model is adapted from [5].
(PDF)

S5 Fig Effect of varying cooperativity and binding affinity (with a reduced *K*_*1*_ –to half its original value). (PDF)

S6 Fig CTB bound as a function of unbound CTB concentration for each of the possible binding states. (PDF)

S1 File Supporting File. (PDF)
