# Supplementary material for: Binding Cooperativity Matters: A GM1-Like Ganglioside-Cholera Toxin B Subunit Binding Study Using a Nanocube-Based Lipid Bilayer Array
Source: PLoS One. 2016 Apr 12;11(4):e0153265. doi: 10.1371/journal.pone.0153265 (PMC4829222; doi:10.1371/journal.pone.0153265)
Supplement: S1 Fig — (a) A TEM image of the silica shell coated onto Ag nanocubes in 2-propanol. (b) A TEM image of the silica shell coated onto Ag nanocubes in ethanol. (PDF) [file pone.0153265.s001.pdf]

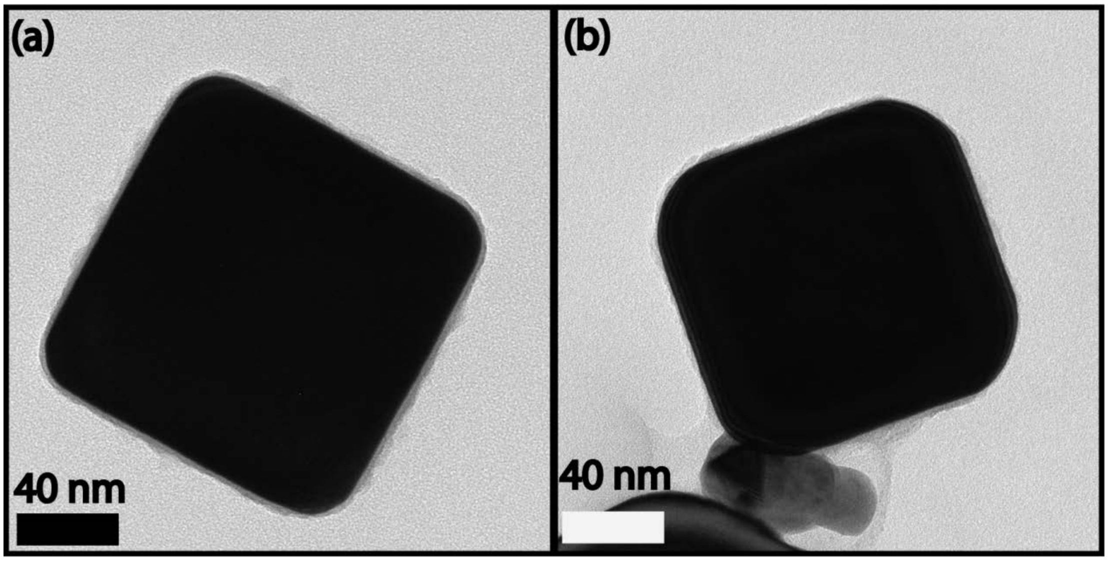

S1 Fig. TEM image comparison of silica coating procedures. (a) A TEM image of the silica shell coated onto Ag nanocubes in 2-propanol. (b) A TEM image of the silica shell coated onto Ag nanocubes in ethanol.
