# Supplementary material for: Binding Cooperativity Matters: A GM1-Like Ganglioside-Cholera Toxin B Subunit Binding Study Using a Nanocube-Based Lipid Bilayer Array
Source: PLoS One. 2016 Apr 12;11(4):e0153265. doi: 10.1371/journal.pone.0153265 (PMC4829222; doi:10.1371/journal.pone.0153265)
Supplement: S2 Fig — Streptavidin (StP)-biotin-DPPE binding data assuming that all StP is bound to a biotin group resulting in the observed LSPR shift. Data are reported as mean ± S.D. (n = 8). (PDF) [file pone.0153265.s002.pdf]

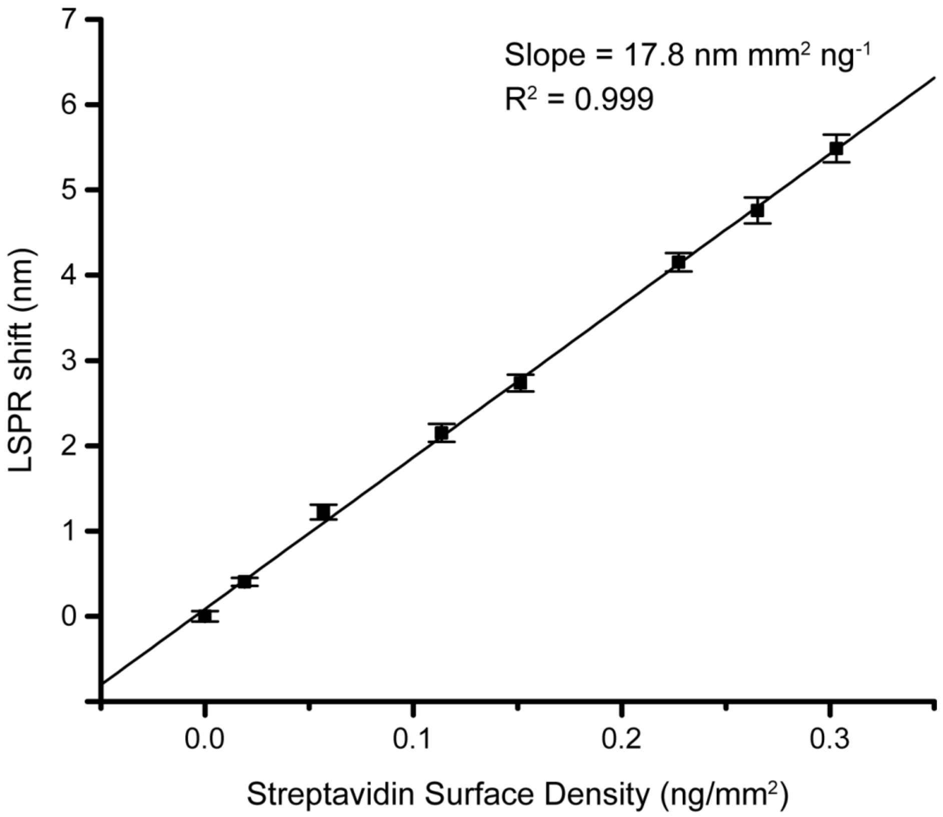

S2 Fig. Equilibrium StP- Biotin binding for sensor calibration. Streptavidin (StP)-biotin-DPPE binding data assuming that all StP is bound to a biotin group resulting in the observed LSPR shift. Data are reported as mean ± S.D. (n=8).
