# Supplementary material for: Binding Cooperativity Matters: A GM1-Like Ganglioside-Cholera Toxin B Subunit Binding Study Using a Nanocube-Based Lipid Bilayer Array
Source: PLoS One. 2016 Apr 12;11(4):e0153265. doi: 10.1371/journal.pone.0153265 (PMC4829222; doi:10.1371/journal.pone.0153265)
Supplement: S3 Fig — Comparison of GM1 gangliosides obtained from various companies with fucosyl-GM1 plotted for reference. Data are reported as mean ± S.D. (n = 8). (PDF) [file pone.0153265.s003.pdf]

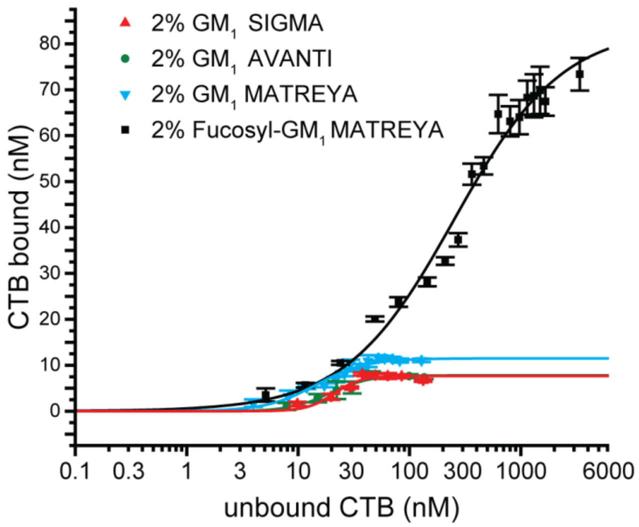

S3 Fig. GM_1 quality comparison across vendors. Comparison of GM_1 gangliosides obtained from various companies with fucosyl-GM_1 plotted for reference. Data are reported as mean ± S.D. (n=8).
