# Supplementary material for: Binding Cooperativity Matters: A GM1-Like Ganglioside-Cholera Toxin B Subunit Binding Study Using a Nanocube-Based Lipid Bilayer Array
Source: PLoS One. 2016 Apr 12;11(4):e0153265. doi: 10.1371/journal.pone.0153265 (PMC4829222; doi:10.1371/journal.pone.0153265)
Supplement: S4 Fig — The stepwise model is adapted from [5]. (PDF) [file pone.0153265.s004.pdf]

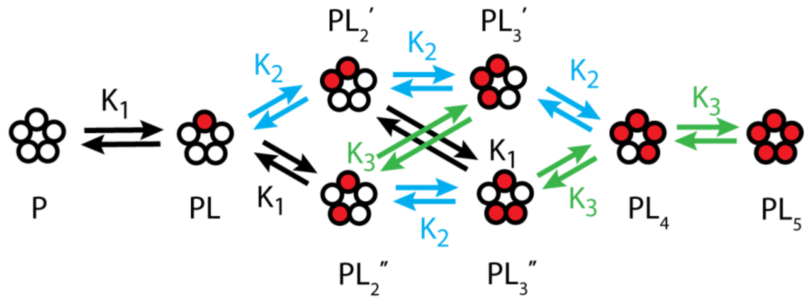

S4 Fig. Stepwise CTB binding model with a single type of ganglioside. The stepwise model is adapted from [5].
