# Supplementary material for: Binding Cooperativity Matters: A GM1-Like Ganglioside-Cholera Toxin B Subunit Binding Study Using a Nanocube-Based Lipid Bilayer Array
Source: PLoS One. 2016 Apr 12;11(4):e0153265. doi: 10.1371/journal.pone.0153265 (PMC4829222; doi:10.1371/journal.pone.0153265)
Supplement: S5 Fig — (PDF) [file pone.0153265.s005.pdf]

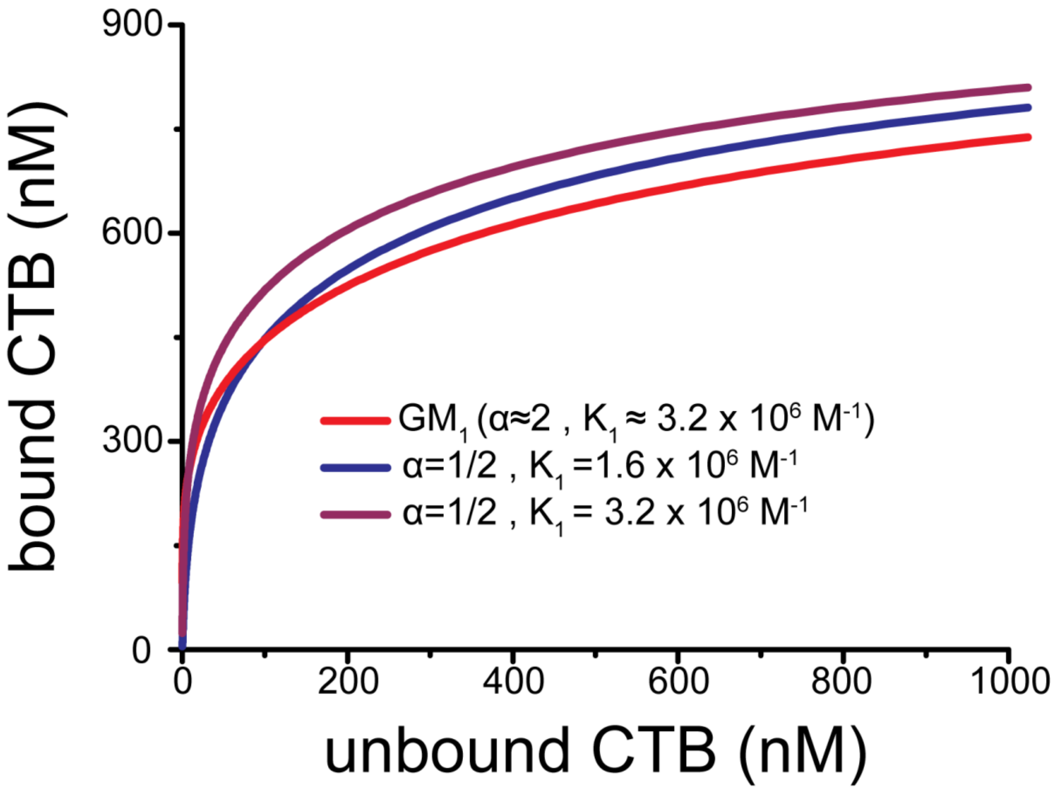

S5 Fig. Effect of varying cooperativity and binding affinity (with a reduced K_1 – to half its original value).
