# Supplementary material for: Binding Cooperativity Matters: A GM1-Like Ganglioside-Cholera Toxin B Subunit Binding Study Using a Nanocube-Based Lipid Bilayer Array
Source: PLoS One. 2016 Apr 12;11(4):e0153265. doi: 10.1371/journal.pone.0153265 (PMC4829222; doi:10.1371/journal.pone.0153265)
Supplement: S1 File — (PDF) [file pone.0153265.s007.pdf]

S1 File

Supporting Information

Binding Cooperativity Matters: A GM_1-like Ganglioside-Cholera Toxin B Subunit Binding Study Using a Nanocube-Based Lipid Bilayer Array

Theoretical Model.

A model for the binding of GM_1 to CTB was proposed by Klassen and his coworkers. [1] (**S4 Fig**). We adapted and analyzed their stepwise binding model with minor changes. All equations are summarized below. Writing the material balance for a pentameric protein P,

$$\mathbf{P_T = [P] + [PL] + [PL_2'] + [PL_2''] + [PL_3'] + [PL_3''] + [PL_4] + [PL_5]} \quad (1)$$

where P_T is the total concentration of protein, [P] is the concentration of free protein, and $[PL]/[PL_2]/[PL_3]/[PL_4]/[PL_5]$ are the protein-ligand binding complexes with 1, 2, 3, 4, and 5 ligands/receptors respectively. $[PL_2]$ and $[PL_3]$ contain two bound states ($[PL_2']$/ $[PL_2'']$ and $[PL_3']$/ $[PL_3'']$) that were defined by Klassen's model. [1]

Writing material balance for the ganglioside L,

$$\mathbf{L_T = [L] + [PL] + 2([PL_2'] + [PL_2'']) + 3([PL_3'] + [PL_3'']) + 4[PL_4] + 5[PL_5]} \quad (2)$$

Where L_T is the total concentration of ganglioside and L is concentration of unbound gangliosides. At equilibrium, the material balances for each reaction species were written as follows:

$$\mathbf{[PL] = 5K_1[P][L]} \quad (3)$$

$$\mathbf{[PL_2'] = K_2[PL][L] = 5K_1K_2[P][L]^2} \quad (4)$$

$$\mathbf{[PL_3'] = K_2[PL_2'][L] = 5K_1K_2^2[P][L]^3} \quad (5)$$

$$[\mathbf{PL_4}] = \mathbf{K_2}[\mathbf{PL'_3}][\mathbf{L}] = \mathbf{5K_1K_2^3}[\mathbf{P}][\mathbf{L}]^4 \quad (6)$$

$$[\mathbf{PL_5}] = \frac{\mathbf{K_3}}{\mathbf{5}}\,[\mathbf{PL_4}][\mathbf{L}] = \mathbf{K_1K_2^3K_3}[\mathbf{P}][\mathbf{L}]^5 \quad (7)$$

$$[\mathbf{PL''_2}] = \mathbf{K_1}[\mathbf{PL}][\mathbf{L}] = \mathbf{5K_1^2}[\mathbf{P}][\mathbf{L}]^2 \quad (8)$$

$$[\mathbf{PL''_3}] = \mathbf{K_2}[\mathbf{PL''_2}][\mathbf{L}] = \mathbf{5K_1^2K_2}[\mathbf{P}][\mathbf{L}]^3 \quad (9)$$

S1 and **S2 Eq** then become:

$$\mathbf{P_T} = [\mathbf{P}] + \mathbf{5K_1}[\mathbf{P}][\mathbf{L}] + \mathbf{5K_1K_2}[\mathbf{P}][\mathbf{L}]^2 + \mathbf{5K_1^2}[\mathbf{P}][\mathbf{L}]^2 + \mathbf{5K_1K_2^2}[\mathbf{P}][\mathbf{L}]^3 + \mathbf{5K_1^2K_2}[\mathbf{P}][\mathbf{L}]^3 + \mathbf{5K_1K_2^3}[\mathbf{P}][\mathbf{L}]^4 + \mathbf{K_1K_2^3K_3}[\mathbf{P}][\mathbf{L}]^5 \quad (10)$$

$$\mathbf{L_T} = \mathbf{L} + \mathbf{5K_1}[\mathbf{P}][\mathbf{L}] + \mathbf{2}\left(\mathbf{5K_1K_2}[\mathbf{P}][\mathbf{L}]^2 + \mathbf{5K_2^2}[\mathbf{P}][\mathbf{L}]^2\right) + \mathbf{3}\left(\mathbf{5K_1K_2^2}[\mathbf{P}][\mathbf{L}]^3 + \mathbf{5K_1^2K_2}[\mathbf{P}][\mathbf{L}]^3\right) + \mathbf{4}\left(\mathbf{5K_1K_2^3}[\mathbf{P}][\mathbf{L}]^4\right) + \mathbf{5K_1K_2^3K_3}[\mathbf{P}][\mathbf{L}]^5 \quad (11)$$

Klassen and his coworkers [1] determined the values for K_1, K_2, and K_3 for CTB binding with GM_1 to be $3.2 \times 10^6\ M^{-1}$, $5.5 \times 10^6\ M^{-1}$, and $9.5 \times 10^6\ M^{-1}$ respectively. To account for cooperativity, we multiplied K_2 and K_3 with a factor 'α'

$$\boldsymbol{K_2 = \alpha \times K_1\,,\, K_3 = \alpha^2 \times K_1} \quad (12)$$

α is approximately 2 when GM_1 is the binding receptor and α <1 represents negatively cooperative binding. From the fitting results of the Hill-Waud equation, we found the binding cooperativity of CTB to fucosyl-GM_1 was significantly reduced from CTB to GM_1 binding; hence, we considered that the value of α to be less than 2. Although we selected an arbitrary α = 1/2 to demonstrate the enhanced binding capacity in **Fig 4a**, the increase of binding capacity was

observed for all tested values of α smaller than the 2, representative of GM_1 binding to CTB. For all theoretical modeling, we set the total ganglioside concentration initially to 10^{-6} M (A similar order of magnitude as the experiments performed), and changed the concentration of unbound CTB from 10^{-10} to 10^{-6} M , and then we solved **S11 Eq** to obtain [L]. Therefore, we could determine the concentration of the bound CTB by adding **S3 Eq** to **S9 Eq.**

References

1. Lin H, Kitova E, Klassen J. Measuring Positive Cooperativity Using the Direct ESI-MS Assay. Cholera Toxin B Subunit Homopentamer Binding to GM1 Pentasaccharide. J Am Soc Mass Spectrom. 2014;25(1):104-10. doi: 10.1007/s13361-013-0751-5.
